# Using the WHO building blocks to examine cross-border public health surveillance in MENA

**DOI:** 10.1186/s12939-025-02393-7

**Published:** 2025-02-06

**Authors:** Laura Buback, Shayanne Martin, Esbeydy Pardo, Farah Massoud, Jesus Formigo, Atousa Bonyani, Noha H. Farag, Zayid K. Almayahi, Kenta Ishii, Susie Welty, Dana Schneider

**Affiliations:** 1 University of California San Francisco (UCSF), San Francisco, USA; 2 U.S. Centers for Disease Control and Prevention (CDC), Middle East & North Africa Regional Office, Muscat, Oman; 3 Centers for Disease Control and Prevention (CDC), Atlanta, USA

**Keywords:** Border health, Cross-border surveillance, Middle East, North Africa, Health systems strengthening, Mobile populations, Mass gatherings, Travel health

## Abstract

The introduction of the Sustainable Development Goals by the United Nations has set a global target for achieving Universal Health Coverage, requiring resilient health systems capable of addressing public health emergencies and ensuring health security. Public health surveillance, crucial for detecting and responding to infectious disease outbreaks, is key to building health system resilience. Due to the high levels of mobility and political instability in the Middle East and North Africa (MENA) region, unique challenges arise in cross-border health surveillance. This review aims to highlight the importance of cross-border public health surveillance in strengthening health systems across MENA to achieve equitable health outcomes.

A mixed-methods approach was utilized, combining a systematic literature review with semi-structured in-depth interviews (IDIs) involving 28 stakeholders from seven MENA countries. The literature review adhered to PRISMA guidelines, while the IDIs provided qualitative insights into current surveillance practices and challenges. Findings from the literature review and IDIs were triangulated and analyzed using the WHO Health Systems Strengthening (HSS) Building Blocks Framework to identify key challenges and recommendations for improving cross-border surveillance.

Results indicate that existing cross-border surveillance systems in MENA face challenges in data collection, analysis, and sharing, with disparities across countries based on income levels and political contexts. Key challenges include delayed and incomplete data sharing, insufficient funding across sectors, inadequate training, inconsistent data definitions, and limited integration of health data for mobile populations. Recommendations emphasize strengthened governance and leadership to facilitate regional cooperation and information sharing, sustainable financing for implementing a One Health approach, utilizing innovative information systems, workforce development to enhance data collection and analysis, and secure supply chains for medicines and vaccines and equitable service delivery for all mobile populations.

In conclusion, the WHO HSS Building Block Framework provides a comprehensive approach to assessing and improving cross-border public health surveillance and enhancing health security and equity in MENA. Strengthening cross-border surveillance systems may help MENA countries meet IHR requirements, achieve greater health security, and advance health equity among all types of mobile populations. Despite limitations, the study offers critical insights for improving cross-border surveillance strategies in the region.

**Supplementary Information:**

The online version contains supplementary material available at 10.1186/s12939-025-02393-7.

## Introduction

Following the introduction of the Sustainable Development Goals (SDGs) by the United Nations in 2015, countries are expected to progress towards Universal Health Coverage (UHC) by 2030. Health systems play a pivotal role in implementing UHC by addressing the six building blocks of the World Health Organization (WHO) Building Block Health Systems Strengthening (HSS) framework: service delivery, access to medicines and health technology, health workforce, health financing, health information systems, and health governance [1]. To effectively achieve UHC and overall health outcomes of SDG three, “ensure healthy lives and promote well-being for all at all ages,” countries should prioritize health system resilience to ensure uninterrupted access to healthcare for their populations during public health emergencies and to maintain health security. This would enable the health systems to effectively mitigate, prepare for, respond to, and recover from public health shocks [2]. In this regard, public health surveillance plays a crucial role in health systems resilience through preventing and controlling communicable and noncommunicable diseases. WHO defines public health surveillance as “the continuous and systematic collection, orderly consolidation and evaluation of pertinent data with prompt dissemination of results to those who need to know, particularly those who are in a position to take action” [3]. Effective surveillance systems help detect infectious disease outbreaks early, allowing rapid response and minimizing disease morbidity and mortality.

Increased global travel and trade involving animals and animal-based foods, as well as human activities and their environmental impact, have contributed to the increased transmission and rapid spread of infectious diseases [4–7]. As a result, epidemics and pandemics are expected to become more frequent, presenting complex challenges for the field of public health [8]. Effectively identifying and responding to health threats requires cooperation across borders. Given the important link between disease spread and human and animal mobility [4–6], cross-border public health surveillance is critical for preparation, early detection, and effective response to potential health threats. Recent literature indicates that public health efforts must consider institutional dynamics and multisectoral strategies, including the One Health approach, in order to improve cross-border health surveillance and pandemic preparedness [9–13]. To increase pandemic preparedness, resilience, and global health security across all countries, it is important to examine cross-border health and surveillance, with an effort to understand existing systems' successes and challenges at the national level and along corridors of mobile populations.

Mobile populations can pose significant challenges for national disease control and prevention.

These populations comprise business travelers and tourists, internally displaced persons, nomadic groups, and international migrants, including labor migrants, refugees, asylum seekers, and climate refugees among others [14]. Each of these groups faces a unique set of health risks and vulnerabilities such as pre-existing conditions, endemic diseases in host countries, environmental exposures, and socioeconomic inequalities [15]. The Middle East and North Africa (MENA) region is of particular interest, as it hosts 2.4 million refugees, 12.6 million internally displaced persons, 251,800 asylum seekers, 370,300 stateless persons, and more than 24 million migrant workers [16, 17]. In addition, it is a major destination for travelers for tourism and work, as well as for mass gatherings, with 128 million arrivals to the region in 2019 alone including 2,500,000 Hajj pilgrims in Saudi Arabia from over 180 countries [18, 19]. The MENA region faces political and economic instability, which limits its capacity to collect health information for all these mobile populations. A recent study showed that infectious disease surveillance data for refugees in particular, despite their growing numbers, is limited [20]. The gap may partially stem from the involvement of multiple partners, the lack of a sustainable systematic data collection approach, and the absence of guiding policies [20].

This review aims to describe the role of cross-border public health surveillance in strengthening health systems across MENA to generate more equitable health outcomes and meet the International Health Regulations (IHR) [21].We conducted a mixed methods review to describe and analyze the collection, analysis and sharing of public health surveillance information within communicable disease surveillance systems in the MENA region. We focus on differences across types of mobile populations and countries, particularly in their income levels and their emergency and routine practices. We use the WHO HSS Building Blocks Framework to identify gaps and opportunities to improve cross-border surveillance and thus, global health security and UHC in MENA. We will demonstrate that surveillance systems should be aligned with each of the health system building blocks. Strong governance fosters international cooperation and adherence to regulatory frameworks such as the IHR. We will describe how sustainable health financing supports the continuous operation of surveillance systems across sectors; advanced health information systems enable efficient management and data sharing; a well-trained health workforce is critical for accurate data collection and analysis; access to essential medicines and technologies supports prompt responses to detected threats; and robust service delivery ensures that health facilities can provide timely data on disease outbreaks. The Joint External Evaluation (JEE) [22] process assesses a country's capacity to prevent, detect, and respond to public health threats in accordance with the IHR. The comprehensive nature of the WHO HSS framework supports the JEE by providing a structured approach to examine health system capacities, including surveillance systems, which are a core component for timely and effective detection and response mechanisms. Inadequate cross-border surveillance systems lead to delayed or inadequate responses to disease outbreaks, resulting in excess morbidity and mortality.

## Methods

### Research design

The data presented in this paper are part of a larger mixed-methods study that explored cross-border, regional, national, and points of entry (POE) surveillance in MENA, along with the contextual factors influencing movement within the region. We used a mixed-methods approach to explore approaches and functionality of data collection, analysis, and sharing. We started with an extensive examination of existing literature and, based on the findings, we conducted in-depth interviews (IDIs) with key stakeholders across the MENA region. For the purposes of this study, we used the U.S. Centers for Disease Control and Prevention (CDC)’s definition of “cross-border surveillance,” which is “the contribution of surveillance data by two or more neighboring or regional countries into each other’s or an intergovernmental agency’s reporting system with the intention of maintaining simultaneous situational awareness of each country’s public health epidemiologic situation and the overall intention to foster partnership, build collaboration, and mitigate the international spread of public health threats.” We also used the CDC working definition of “mobile populations”: “Mobile population” is an inclusive term that refers to all groups of people who are on the move, whether for short or long distances or short or long durations. Mobile populations include travelers (business and recreational), individuals voluntarily relocating either temporarily or permanently to a new location (e.g., students, expatriates, military personnel, development workers, immigrants), and forced migrants (e.g., internally displaced persons, refugees).

### Literature review

We conducted a literature review, adhering to Preferred Reporting Items for Systematic Reviews and Meta-Analyses (PRISMA) 2020 [23] guidelines, to assess existing studies and synthesize information relevant to cross-border, regional, national, and POE surveillance for mobile populations in the MENA region.

#### Search strategy

Thirteen databases, including PubMed and Google Scholar, were searched for peer-reviewed and grey literature. Search terms included MENA countries, and specific key terms related to cross-border surveillance, mobile populations, point of entry, and border health were employed. Search results were filtered based on publication dates to ensure relevance. After this initial review we refined search parameters focusing on emerging key terms.

#### Inclusion criteria

We included the 24 countries listed by WHO EMRO, World Bank MENA Region, or the Organization for Economic Co-operation and Development (OECD) MENA Region.^1^ Publications were included if it reported on at least one MENA country and involved movement in or out of the MENA region. Publications from all databases were examined to ensure they were not older than 2012. For publications sourced from Google Scholar, the criteria were not older than 2018, due to the extensive search results in this database and time limitations of the research team.

#### Data collection and synthesis

Four independent reviewers assessed the articles for inclusion and categorized them based on their respective topics (LB, SM, EP, JF). Matrices and findings from each category were compiled into a single document for a comprehensive review. Information was extracted from selected articles, including article details, countries included, key themes, challenges and recommendations. A narrative approach was used to synthesize and analyze the extracted data. Findings were summarized in tables and presented in a thematic synthesis. Tables, results, and findings have been reported comprehensively elsewhere (manuscript in progress).

### In-depth Interviews (IDIs)

We used IDIs to explore key stakeholder perspectives of mobile populations, cross-border surveillance systems, International Health Regulation (IHR) implementation, multi-sectoral collaborations, and One Health in the MENA region.

#### Participant selection

Using purposive sampling, we selected stakeholders with direct experience in border health or health surveillance in the MENA region. We aimed to include countries in the Gulf region, as well as North Africa, high- and low-income countries and ensured inclusion of conflict affected countries. Seven of nine countries invited to participate accepted. The US-CDC and WHO facilitated the introduction of the project and interviews, either through the Ministry of Health or WHO Country Office. Outreach to countries was conducted by email, sharing the Information Sheet in Annex 1. Of the 60 participants invited, 28 agreed to participate in stakeholder interviews. Reasons for refusal included no response and heavy workload/lack of time. Seven represented regional stakeholders working across various countries in MENA and 21 national stakeholders represented seven counties (Table 1). Countries were selected to provide a variation of case studies across geographical and country income level variations.

**Table 1:** In-depth interviews

Country	Income level	Number of Interviews	Gender Distribution	Organizations	Work Function/Position National/Provincial/Other
Libya	Upper Middle	1	1 Male	National Center for Disease Control	1 National
Morocco	Lower Middle	2	2 Male	Ministry of Health	2 National
Oman	High	6	1 Female, 5 Male	Ministry of Health, Ministry of Agriculture, Civil Aviation Authority	3 National, 3 Provincial
Saudi Arabia	High	4	2 Female, 2 Male	Ministry of Health	2 National, 2 Provincial
Sudan	Low	4	1 Female, 3 Male	Ministry of Health, EMPHNET, WHO	1 Consultant, 2 National, 1 Other
United Arab Emirates (UAE)	High	3	3 Male	Ministry of Health	3 National
Yemen	Low	1	1 Male	WHO Health Sector	Other
Regional Organizations		7	2 Female, 5 Male	WHO, EMPHNET, UNRWA, IOM, Gulf CDC	
Total		28			

#### Data collection

Data were gathered through in-depth, semi-structured interviews, either in English or Arabic. An interview guide, informed by the literature review, covered topics such as definitions of mobile populations, surveillance systems, IHR implementation, multisectoral collaborations, One Health, and case studies (Annex 2). Interviews were conducted either in-person or via online call, lasted 60–90 min, and were audio-recorded following verbal consent. Interview audio recordings were transcribed using Temi transcription software [24] and Arabic interviews were translated into English using ChatGPT [25] and reviewed by a native Arabic speaker fluent in English (FM).

#### Data analysis

We used a combination of rapid matrix [26] and thematic analysis [27] techniques. This rapid qualitative method has been proven to be useful in resource-constrained contexts to promote timely uptake of findings by implementers [28, 29] and was most applicable to our time and resource-constrained settings. Five research team members summarized the interview transcripts using a predefined template of research domains (LB, SM, EP, FM, JF). The key summary points were entered into that were entered into REDCap [30] electronic data capture tools hosted at UCSF. REDCap facilitated matrix generation organized by key themes. We thoroughly reviewed the summaries and matrices to identify the key points, approaches, challenges, and recommendations.

#### Ethical considerations

The study received ethical approval from the University of California, San Francisco (UCSF) Institutional Review Board (IRB, study number: 22–37,958). This activity was reviewed by CDC and was conducted consistent with applicable U.S. federal law and CDC policy. Both boards deemed the study exempt from collecting personal health information (PHI). The participants were invited to participate by email, including an information sheet explaining the purpose of the study and plans for use and dissemination of information (Annex 1). Participants accepted participation by email and provided verbal informed consent for recording of interviews. Participants were also notified that they would be informed of study results and recommendations.

### Triangulation

The research team reviewed and triangulated findings from the literature review and IDIs, comparing key challenges and recommendations for each theme and synthesizing around the health systems strengthening building blocks. To evaluate the overlap between the literature and interview findings, the interview template was designed to track recommendations identified in the literature review, assessing how frequently these recommendations were mentioned or implemented by stakeholders during the interviews. In Table 2, we demonstrate the extent that recommendations from the literature review were discussed in IDIs, noting how many countries and regional stakeholders mentioned an intervention, and if the recommendation is reportedly implemented. The UCSF Artificial Intelligence platform Versa AI (2024 Version) [31] was used to summarize some of the key findings presented in this review. Long elements of summarized text were entered in Versa AI to produce shorter syntheses related to key findings. No interview transcriptions or personally identifiable information were entered into the Versa AI, however Versa AI ensures data protection. Versa AI keeps all data inside UCSF and complies with all HIPAA requirements. This is an approved platform for use with all UCSF data and guarantees data protection through strict access controls, employee training, and clear data retention policies. No information is saved by any third parties. Together, these measures uphold user data’s privacy and security. While no personal identifying information (PII) was collected in interviews, the use of Versa AI was a measure to ensure confidentiality of all IDIs.

**Table 2:** Triangulation of Recommendations from Literature Review with IDIs

			**Relevant Building Block**^**a**^					
**Recommendations from literature review**	**Mentioned in interviews**	**Implemented in countries interviewed**	**BB.1**	**BB.2**	**BB.3**	**BB.4**	**BB.5**	**BB.6**
Establish Memorandums of Understanding/Data sharing agreements between countries to strengthen routine surveillance & mass gatherings	2 countries	6 countries	X					
International adoption of definitions/indicators & interoperable/comparable data sources led by supernational body	1 country 2 regional	3 countries	X		X			
Partnerships across government, international organizations, academia, and private sector to improve cross-border data sharing	1 country	4 countries 2 regional	X					
Enhanced multi-stakeholder engagement at national and international level	6 countries 2 regional	4 countries 2 regional	X	X			X	
Strengthening early warning alert and response (EWAR) surveillance systems within border communities, refugee camps, and informal refugee settlements/Event Based Surveillance	2 countries 1 regional	3 countries			X			X
Optimize resource allocation for One Health implementation at regional and national level	2 countries		X	X		X		
Employ a variety of digital tools and mobile applications to facilitate data collection, especially amongst hard-to-reach populations	1 country 1 regional	1 country 2 regional			X		X	X
Promote more integrated rather than vertical disease surveillance	3 countries 1 regional	2 countries 1 regional			X	X		
Conduct capacity building for national health officials, especially in in data manipulation and new technologies	3 regional	1 country 1 regional			X	X		
Integrate migration data from diverse sources, such as international organizations collecting data on vulnerable populations, into national health information systems	2 countries 1 regional	3 countries 1 regional			X			X
Employ innovations in block chain, vaccine passports, refugee cards, mobile data, and electronic bracelets, to improve quality (accuracy, speed, completeness) of screening and traveler surveillance while maintaining attention to personal privacy and permissions	1 country, 1 regional	1 country 1 regional			X		X	
Cross-border health initiatives (CBHI) and multi-sectoral cross-border committees during emergencies	3 countries	4 countries 2 regional	X					X

^a^1: Governance and Leadership; 2: Health Financing; 3: Information System; 4: Health Workforce; 5: Essential Medicines, Vaccines and Technology; 6: Service Delivery

### Building blocks framework

The WHO describes health systems using six core components also referred to as “building blocks” [32]. These building blocks are: *Leadership and Governance* focuses on management, coordination, and decision-making within the health system; *Health Financing* encompasses how health services are funded, including financial mechanisms and resource allocation; *Health Information Systems* includes health data collection, analysis, and dissemination for informed decision-making; *Health Workforce* involves the availability, distribution, and capacity of health professionals; *Medical Products* ensures that essential medicines, vaccines, and other health technologies are available, accessible, affordable, and of good quality; *Service Delivery* is comprised of the provision of health services to people in need. We used this framework to delineate the components of cross-border surveillance and comprehensive surveillance systems that include all types of mobile populations. This enabled the identification of specific actions to strengthen cross-border surveillance systems.

## Results: Cross-border surveillance from a health systems strengthening perspective

**Fig. 1:**
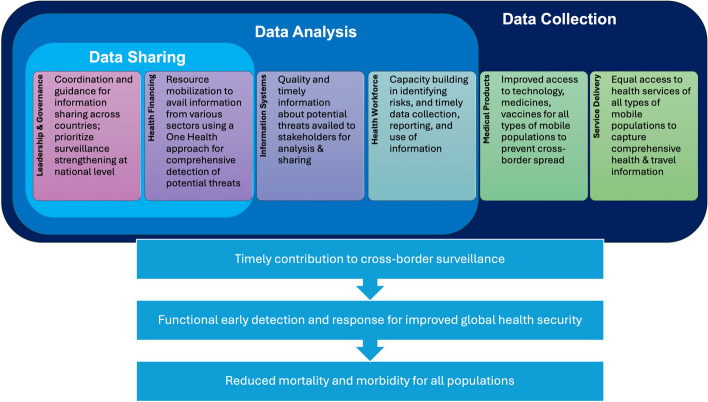
WHO Health System Strengthening Building Block: Components of cross-border surveillance and improved health outcomes

We used the WHO HSS building blocks framework to identify how cross-border public health surveillance in MENA can be assessed and strengthened. Despite approaches differing by countries’ income levels and political contexts, the building blocks provide a unified framework for delineating the strengths and challenges of surveillance components we explored in the study: data collection, data analysis, and data sharing (Fig. 1*)*. The definition of cross-border surveillance is anchored in the contribution of information from individual countries for sharing and collaboration across countries. However, each country must strengthen its collection, management, and analysis of data on all types of mobile populations to improve its ability to detect threats and contribute accurate, reliable, and timely information to its neighbors and partners. Figure 1 shows leadership and governance and health financing are instrumental across all three steps, information systems and health workforce are essential for both data collection and analysis, and service delivery and medical products are most critical for ensuring comprehensive data collection across all types of mobile populations. In this review, we delve into each building block, explaining challenges and recommendations to strengthen each important element of an inclusive, secure, and functional surveillance system for cross-border information sharing. Each of the blocks described below has a path forward for examining and improving cross-border surveillance from a different component of the health system. Table 2 also delineates each recommendation’s application to the HSS building blocks.

### Governance and leadership

Leadership and governance are critical for improving disease information sharing across countries in MENA, both within and across countries. The WHO’s IHR governing framework for global health security includes requirements for information sharing in public health emergencies [21], but our review found implementation was mixed. While the literature highlighted gaps in IHR implementation [33, 34], some IDIs, mostly in higher income countries, reported strong implementation, capacities, and functionality of IHR focal points, with pride of their JEE results. On the other hand, another low-income country noted it had not participated in a JEE process since prior to the COVID-19 pandemic, but suspected its JEE would be an “exercise in building a very, very big list of gaps” (3.E.1). Challenges in IHR and information sharing reportedly persist due to political sensitivities, lack of trust, and operational barriers in implementing IHR and existing governance agreements. These challenges were explained in IDIs, particularly in interviews with regional stakeholders that delved into examples of countries sharing late or incomplete information that resulted in suboptimal management of outbreaks. Ethical and security concerns surrounding health data sharing are critical, leading discussions to focus primarily on aggregated, high-level information or de-identified details about threats or cases. As a result, the term 'information sharing' is more commonly used than 'data sharing' in the MENA region.

Regional governance structures, such as the Khartoum Declaration and Gulf Cooperation Council (GCC), also improve timely data exchange across borders. The example of the Gulf CDC, explained in IDIs, demonstrates how strengthened leadership and governance at a sub-regional level can improve information sharing during public health emergencies. The Gulf CDC, a collaborative network comprising Bahrain, Kuwait, Oman, Qatar, Saudi Arabia, and the United Arab Emirates, aims to enhance information exchange between these six member states. Established in response to challenges in information transparency during the pandemic, Gulf CDC operates as a neutral entity trusted by countries to facilitate amendments to the IHR and agreements between nations. Amid and following the COVID-19 crisis, the organization ensured consistent communication between member states, with designated multisectoral focal points meeting weekly to discuss priority diseases and identified risks. The small size of Gulf CDC, political commitment from the existing GCC, and consistent communication platform have appeared to enable preliminary success, having a positive impact on information sharing relationships with neighboring countries.

The Gulf CDC approach to governance is also noteworthy in its multi-sectoral approach across countries. Effective governance also relies on multi-sectoral collaboration at the national level among stakeholders, including policymakers, healthcare providers, and local communities, for strengthening capacities and coordination [35, 36]. The One Health approach recognizes the interconnectedness of human, animal, both domestic and wild, plant and environmental health and aims to minimize disease threats by mobilizing different sectors to work together [37]. The literature, as well as most participants, agree that adopting a multi-sectoral approach with a One Health Framework is crucial for effectively controlling emerging zoonotic diseases, a strategy being implemented in some capacity by all countries interviewed. Each country interviewed and the literature findings highlighted the vital role of effective communication and collaboration between the human and animal health sectors in addressing disease outbreaks in emergency and conflict settings, particularly among refugees who rely on livestock as their primary source of income [38]. Although a few countries have yet to formally adopt a 'One Health' approach, all have established cross-sector communication and coordination mechanisms, either informally during emergencies or through formalized protocols. Oman and Yemen have adopted a multisectoral approach, which has been instrumental in containing vector-to-human transmission outbreaks. A participant from Saudi Arabia also elaborated on the vital role One Health played during the MERS outbreak in Saudi Arabia [36] and how that has been built upon:

“*[In terms of One Health] we have shared committee with Minister of Agriculture, and we’re still seeking information about their positive cases in camels and our positive case cases in human beings. And actually, now the surveillance team in the regions is a unified surveillance team and a Minister of Agriculture representative there in the region, and a representative from Minister of Health in the region go together and do the surveillance to do activities together.”* (5.A.3)

Similarly, a One Health Focal Point from a low-income country highlights inter-Ministry coordination growing with quarterly meetings for epidemic departments in both ministries:

*“Coordination mechanisms exist, especially between the Ministries of Health and Animal Welfare. This need has been operationalized and institutionalized in recent years. One Health was a challenge because it was working between two ministries, Animal Welfare and Health. In recent times, people have tried to include the third sector, which is the environment, and as we know internationally, there is great interest in the One Health issue. So, [our country], being aware of these instructions, was fully engaged globally in the field of One Health.”* (7.A.2)

Leadership and governance at the international level is thus impacting national multi-sectoral coordination momentum that facilitates information to avail for potential threats and cross-border collaboration.

Regional stakeholders also highlighted the need for continued efforts to operationalize data-sharing agreements and align definitions and indicators, which corroborated the literature [39]. Furthermore, three countries reported working on this. The literature suggests that international and bilateral governance to harmonize indicators and information sharing agreements, as well as expanded partnerships with academia and the private sector, can strengthen information sharing. Given the complex histories of borders and ongoing conflict in the region [40–44], academic and government partnerships were highlighted as an opportunity to bridge political turmoil [45] and strengthen surveillance capacity in vulnerable states, providing an opportunity to strengthen weaker health systems. International collaborations involving politically neutral academic institutions have demonstrated the ability to maintain partnership across countries in turmoil. For example, The Middle East Consortium for Infectious Disease Surveillance (MECIDS) is a regional collaboration of academic and public institutions aimed at facilitating IHR implementation and improving the laboratory capacity that facilitates information sharing across national reference laboratories of neighboring countries Israel, Jordan, and the Palestinian National Authority. [45, 46]. Furthermore, leadership to enhance coordination of public and private sector health facilities [47, 48] in surveillance efforts will improve coverage of surveillance data for all types of mobile populations, exemplified by the case of travel health services coordinated across both sectors in Oman [49].

### Health financing

Governance, leadership, and multi-sectoral coordination to implement information sharing, One Health, IHR, and workforce supports within and across countries relies heavily on sustainable health financing. Sufficient funding for all sectors would improve data collection, analysis, and sharing to adopt a multi-sectoral approach. In particular, sustainable health financing will be critical for implementing a One Health framework to effectively control emerging zoonotic disease with the potential for cross-border spread. The One Health approach addresses health security by focusing efforts on areas prone to emerging threats, including monitoring zoonotic diseases, neglected tropical diseases, and vector-borne diseases. Although participants commonly referenced SARS-CoV-2 (COVID-19) and Middle East respiratory syndrome (MERS-CoV) as diseases of concern in the MENA region, the literature highlighted additional diseases that are high priority for certain countries, with a major demonstration of varying prevalence of vector-borne diseases across countries. As examples, Rift Valley Fever is a major concern in Saudi Arabia, Egypt, and Yemen [50, 51], Chikungunya poses a significant threat in Sudan and Yemen [34, 51, 52], and Dengue is a pressing issue in Oman and Saudi Arabia [51]. Further, Malaria is prevalent in Yemen, Oman, and Saudi Arabia, while Egypt faces a high rate of West Nile and Zika viruses [51].

The amount of funding a country receives also impacts its ability to respond to public health threats. The literature emphasized the financial challenges of meeting the IHR, especially in fragile and conflict-impacted countries. For example, Yemen faces significant financial challenges due to inadequate planning and resource allocation, intensified by its unstable political environment [53]. Although there are many qualified public health professionals capable of developing necessary health guidelines to mitigate the spread of diseases in Yemen, the ongoing conflict makes securing critical financial support challenging because of the lack of trust and willingness from donors and investors [53].

In Libya, on the other hand, a participant stated that their biggest financial challenge the country faces is in human resources:

*“Human Resources is the biggest challenge as well as the finance. We have many, complicated procedures to be followed. To make new contracts, you need finance, and you need some approval from the health ministry, et cetera. That's why there is a challenge.”* (6.A.1)

Financial shortages are not limited to just low-income or conflict-impacted countries. For example, participants in a high-income country, reported financial challenges in human resources:

“*The main challenge we are facing [is] human and financial resources, sometimes it is the economic situation. These financial matters are very difficult to control, however, we work with what we have. We even have shortages in some of the categories for health workers but we’ve been covering for each other. The health sector needs more and more support. The health budget is getting bigger however the spending is more than what is calculated. I think the health sector needs financial and human resources as well as materials.”* (2.A.3)

Overcoming challenges related to sustainable funding across all sectors remains imperative. This includes ensuring adequate funding prioritization and allocation [37] across various ministries and establishing clear mandates for funding cross-cutting activities. The WHO Regional Office for the Eastern Mediterranean (WHO EMRO) comprehensive One Health framework (2022) outlines strategic steps to accelerate the implementation of the One Health approach within the Eastern Mediterranean region [37] and calls for identifying financial needs and possible funding mechanisms and specifies resource mobilization as a key responsibility for the regional One Health Quadripartite of UN organizations (Food and Agriculture Organization/UN Environment Program/WHO/World Organization for Animal Health) coordination mechanism Executive Board. The animal health sector faces significant funding gaps across countries in the region. Participants emphasized several key areas that require attention, particularly in the context of limited funding for multisectoral and One Health initiatives. These challenges include inadequate training for border staff, high turnover rates, and insufficient funds for monitoring animals across borders, particularly those crossing borders with migrants. The variation between the animal health sector and the more developed human health sector hinders the implementation of a comprehensive One Health approach in the region. To strengthen the multisectoral response engagement in the MENA region, it is crucial to prioritize funding and capacity building for the animal health sector. The financing and resource mobilization aspects of One Health are still developing in the region, and sustainable funding for animal surveillance, as well as increased collaboration among sectors, are necessary. By addressing these gaps, the region can enhance its ability to effectively implement or strengthen the One Health response system in the MENA region. To effectively control and contain disease outbreaks, it is vital to sufficiently fund different surveillance domains, including the IHR, human resources, and One Health and other multi-sector surveillance approaches.

### Information systems

Cross-border surveillance relies on health information systems that facilitate data collection and analysis that avails quality, timely, and secure information to decision makers, however these processes and functionalities vary across the MENA region. The literature review highlighted several challenges, including data incompleteness, inconsistent definitions, and difficulties in reaching many mobile populations for data collection, such as travelers, nomads and illegal migrants, especially in emergency contexts [46, 54–59]. Participants also highlighted data quality challenges, whereas a regional participant expressed concern with the inconsistent definitions of varying information systems: *“The different categories affect current cross border surveillance work because if we do not have a homogenous definition it means, especially when we're reporting, we in most cases underreport.”* (0.F.1).

We found evidence of innovative solutions in MENA that can be leveraged to provide reliable and timely data to decision makers on potential health threats. The literature recommended integrating migration health data into national health information systems (HIS) [54], adopting international definitions and indicators [60], and employing innovative methods like mobile applications, blockchain technology, geospatial mapping of human mobility, as well as respondent-driven sampling (RDS) to ensure reach of vulnerable populations who miss interaction with the formal health system, especially in emergency situations. Opportunities demonstrated include the potential of using mobile phone data to predict dengue outbreaks [61] and existing labor and household surveys to predict COVID-19 hotspots in Pakistan [62]. While technological advancements may facilitate more timely, complete, and better-quality data, considerations for personal privacy and the right to information protection need to be accounted for in deploying and communicating about new technologies [63, 64].

National surveillance systems that primarily draw information from health facilities are one of the main sources of information for countries. All countries interviewed reportedly had an electronic surveillance system, but these varied in scope and strength, as well as the importance of strengthening health facility information systems to capture data on mobile populations, as exemplified by the electronic surveillance systems in the Gulf region countries. The recommendations mentioned in the literature were not universally recognized in the perspectives of key stakeholders, even though the challenges identified were similar. Innovations and integrated electronic systems were most discussed and implemented by Saudi Arabia and Oman, where the governments have invested more in new technologies, as shown in Table 2. Regional-level stakeholders that work in surveillance also cited similar recommendations but did not necessarily have evidence of implementation. This suggests that further dissemination of existing innovations and resources in technology, data integration, and capacity building be shared with key stakeholders in MENA to provide additional ideas to resolve their challenges, with an attention to the low-income countries and conflict settings to ensure information systems are robust to include, but also protect the privacy of, health and mobility data on all populations, regardless of mobility status, in all countries in the region.

Ethical considerations in cross-border surveillance are critical to ensuring that the collection and use of health data respect individual privacy, minimize harm, and protect vulnerable populations who require additional consideration to ensure their health data is properly collected, stored, and shared. The WHO data-sharing policy provides a framework for international data sharing, emphasizing the anonymization of data to protect individual identities and prevent stigmatization or exclusion of populations [65]. Adopting such practices enables cross-border surveillance systems to address unique challenges effectively while safeguarding individual rights and are an important component of strengthening information systems.

### Health workforce

Health Workforce underscores the need for surveillance systems to be implemented by well-trained, apportioned personnel who can effectively execute data collection and analysis. With increased globalization putting pressure on the public health workforce, members of the health workforce and other sectors (e.g., aviation, commerce) need to be trained in surveillance measures, specifically health facility staff, sub-national and national level surveillance staff, and POE staff. Workforce challenges were a common theme in both the literature and interviews and can be summarized into three challenge areas. First, inadequate training causes personnel to lack understanding and technical capacity to perform their roles [66–68]. Critically, the lack of trained personnel impairs the quality of data collection, timeliness of reporting, and underreporting of disease cases. Second, workforce supply issues caused by high turnover, absenteeism, and staff shortages result in incomplete detection of ill mobile populations, data reporting, and supervision. In both low- and high-income countries in MENA, POE are especially understaffed, due to their nature of being in remote and challenging environments to live. Low-income countries are further challenged by interruptions in pay and low salaries.

A participant from a low-income country shared the challenges in facing significant disruptions during the rainy season due to the inaccessible borders and communication breakdowns. To address these challenges, stakeholders integrated POE into sentinel site networks during emergencies and implemented a zero-reporting system to ensure consistent monitoring. A participant from a high-income country in the health sector also emphasized that the importance of surge capacity in the emergency context, often exacerbating human resource challenges:

“*It always depends on the emergency situation. For instance, you usually build your capacity at a normal level, but it varies from one challenge to another. For example, during the COVID-19 pandemic, there were challenges in terms of hospital capacity, such as available beds. The capacity of the human resources is a key factor.*” (2.G.1)

Staff shortages in other sectors involved in public health surveillance (e.g., agriculture, animal welfare) also impede public health surveillance, especially with a One Health approach. Third, coordinating personnel across multiple sectors and government levels, essential to One Health, is complex, resulting in delayed responses to outbreaks.

Solutions for addressing workforce challenges emerged from both literature and some IDIs. Target areas for training were identified as One Health [66–68], statistics/population dynamics and geospatial mapping [59], genomic epidemiology [69], and multi-country collaboration [33, 69, 70]. In tandem, adequate pay and incentives are needed to retain staff. Otherwise, resources used for training are wasted, and it becomes harder to coordinate teams who are unfamiliar with each other and operating procedure. Better coordination between state, federal and regional actors was highlighted to improve outbreak preparedness and response [22, 46, 71, 72]. Training personnel from other sectors or community leaders and volunteers and well-coordinated task sharing can help full surveillance workforce gaps [42, 73]. As demonstrated by Sudan in border regions, Community Event-Based Surveillance (CEBS) involves community leaders and volunteers who are trained on public health methods to detect and report notifiable diseases. Sudan trained more than 6000 volunteers for CEBS, and has supported the country significantly, although serious communication and implementation challenges resulted from the ongoing conflicts/wars [74, 75]. CEBS is a key surveillance strategy to utilize community level workforce to expand coverage of human resources available for early detection and response to disease outbreaks [76]. A One Health expert from Sudan emphasized the effectiveness of CEBS, stating:

“*If you were to ask me what I consider effective in surveillance systems, I think community-based surveillance is a highly successful system as it supports the primary surveillance system. I train community volunteers in specific locations on specific packages, and they enhance reporting, bringing significant changes in reporting, addressing many gaps that might exist without such systems.”*(7.A.2)

This can potentially be applicable throughout MENA regardless of country income level, including those with emergency, conflict, and migratory settings.

### Essential medicines, vaccines and technology

Strengthening cross-border surveillance includes ensuring the availability and accessibility of essential medicines, vaccines, and technology for mitigating spread of disease across borders. This is crucial for all types of mobile populations for prevention, detection, and response intervention, monitoring, and data collection. MENA countries also must consider special attention to disease surveillance among travelers and attendees at mass gatherings, due to the potential for mass spread. Challenges in this area include maintaining a secure and reliable supply chain for medicines and vaccines, addressing data quality issues, and implementing advanced technologies for effective surveillance [64, 77]. High-income countries like those in the GCC often have better resources and technology to manage these challenges compared to low-income countries, which struggle with outdated guidelines and limited resources. For example, Saudi Arabia's investment in traveler surveillance through health clinics and the use of blockchain with end-to-end encryption for secure data management to ensure integrity and privacy of testing and vaccine information of travelers [78, 79] illustrates the potential benefits of advanced technology and robust infrastructure in improving health outcomes and disease control [56, 80, 81].

A Ministry of Health infectious disease expert from Saudi Arabia highlighted their approach to addressing the unique challenges posed by mass gatherings, stating:

*“Yet, we have the crowdedness, and we have the mass gathering in Ramadan and also in Hajj. So, what do we do to, to face all that? The first one is to increase the capacity of our healthcare workers in the point of entry, either the number of healthcare workers. As we do in each Hajj and each Umrah season, we add more healthcare workers to the point of entries. And instead of having one shift or two shifts, now we work for three shifts in all points of entry for all healthcare workers. And also we increase the training sessions for the healthcare workers around the kingdom."* (5.A.3)

This approach demonstrates how investments in workforce capacity and training can enhance preparedness and response.

Recommendations emphasize the need for multi-stakeholder engagement, improved cross-border communication, and the use of innovative technologies to enhance surveillance and data collection [45]. Deploying digital tools and technologies such as mobile applications, electronic wrist bracelets to provide real time data during mass gatherings, and blockchain can improve the accuracy and security of data on testing, treatment, and vaccinations, particularly for managing large volumes of travelers during events like Umrah and Hajj [77–79, 82]. The Ministry of Health representative highlighted the use of such innovations, stating:

“*In the last Hajj, which is almost months ago, we used the bracelet for the Hajj, which contains the electronic bracelet. It has all the information about the Hajj and his or her health status, chronic diseases, or comorbidities. Also, those data can be easily obtained from those systems to one platform, which is the Hajj information system*."(5.A.3)

Successful implementations include Saudi Arabia's integration of electronic bracelets and traveler health information into the national surveillance system, described by the Saudi Arabia participants, Egypt's monitoring of respiratory and arboviral viruses among pilgrims [83], and the use of mobile application to collect data about vaccinations to support Syrian Refugees in Zaatari Camp in Jordan [84, 85]. Each data capture method must be applicable to the security concerns of its relevant target population. Strengthening these supply systems with technology requires international support and collaboration to ensure timely supply of essential medicines and vaccines, and the development of standard operating procedures for rapid response during public health emergencies. These efforts are essential for mitigating spread of disease amongst all types of mobile populations across borders.

### Service delivery

Improved Service Delivery in the context of cross-border surveillance emphasizes the importance of healthcare access for all types of mobile populations who are often excluded from health monitoring. This in turn improves detection of new diseases and health threats. Yet in the MENA region, the challenges are acute due to climate change, political instability, and mass displacement [38, 43, 86, 87]. Low-income countries face challenges due to limited resources and healthcare infrastructure, leading to disparities in addressing health impacts, including those related to climate change. A regional stakeholder described the link of improved access to primary healthcare for all and improved surveillance and response:

*“Regardless of your nationality, regardless of your migration status, you should be able to access healthcare. So, I think this would help because sometimes people fall ill and they're scared of going to the hospital because they fear they may be turned in cause they do not have the right documents. But then if these access is given to everyone and people can easily access healthcare services at all levels, be it at points of entry or just in the community, we would be able to capture any disease before it spreads… rather than wait for an outbreak of a disease to occur at some particular center where we have a group of mobile populations…So if we do increase access to healthcare services to everybody, this would go a long way in trying to address some of the challenges that would come if people do not access healthcare.”* (0.F.1)

Thus, ensuring surveillance data is available for certain vulnerable populations enables early response to potential outbreaks which subsequently reduces morbidity and mortality amongst these groups. However, refugees and other IDPs face unique challenges, such as continued movement and undocumented status, which may hinder the collection and accessibility of health information as stated by a health official in Yemen: “*One major gap, I guess, for refugees is that, I guess they keep moving. And of course, because of the illegal status…I guess it would make it difficult just to access the information.”* (3.E.1).

The literature and interviews further emphasize the importance of comprehensive data collection and integration of health data for refugees, internally displaced persons (IDPs), and migrants into national systems [54]. Key recommendations from the literature review include implementing cross-border health initiatives and expanding travel health services, which integrate disease surveillance into healthcare systems [49, 71]. Due to the high volume of international travel for business and religious tourism in MENA, travel clinics are useful for providing health education, vaccines, and other preventive care [49]. Enhanced surveillance systems, like EWARS, primary healthcare facilities and mobile clinics supported by international organizations like IOM [88], and digital tools, such as mobile health applications, are essential for improving health service delivery and equity in these contexts [42, 84, 85, 89, 90]. For example, The United Nations Relief and Works Agency for Palestine Refugees (UNRWA) provides healthcare to nearly 5.9 million registered Palestine refugees through 140 clinics using an electronic medical records (EMR) system [91]Specific strategies, such as using digital tools for real-time health information dissemination and establishing electronic health records, are highlighted for their effectiveness in improving service delivery. Countries hosting Palestinian refugees demonstrate how these approaches can enhance disease surveillance and healthcare delivery. As described by an UNRWA representative:

“*We have electronic medical records and we provide primary healthcare in the 140 clinics in these five different places (Gaza, West Bank, Lebanon, Jordan, and Syria), and all of them are connected by internet and electronic medical records. It contains all the services we provide at health centers based on the refugee registration system, and it contains the health data, the maternal child health data, diabetes, hypertension data, outpatient data, and all the others*." (0.I.1)

This digital platform supports efficient information exchange and accurate data collection through automated data checks, encompassing primary care, maternal and child health, chronic conditions, outpatient visits, and communicable diseases. Addressing the challenges of movement across porous border areas, incomplete data, and limited inter-agency coordination is crucial for improving health services for all mobile populations in MENA countries, thereby ensuring health equity [54, 71, 92].

### Limitations

Our findings have limitations. First, only English language searches were conducted, thus excluding anything in Arabic or French, common languages of the region. Furthermore, the gray literature and policy documents on the topic are expansive and we did not have access to all country reports and policy documents throughout the region, especially those in languages other than English. Thus, the findings are biased towards the English language, and those published online or in scholarly journals. Second, interviews were conducted with only seven countries in the region, and cannot be considered representative of all countries, but instead as key insights from specific contexts. The research team comes from the health sector and therefore there is some selection bias towards participants in the health sector, compared to those in animal health or other sectors. Those that responded to the invitation and volunteered to participate may be pre-disposed to a previous work and interest in the topic, leading to a selective participation bias. Different themes may have been found among the non-responders. The is also a possibility of social desirability bias in the interviews, as interviews were conducted by a research team that was funded by the US-CDC, who is a partner involved in public health surveillance throughout the region. Nevertheless, the lessons we gathered are critical to consider when moving forward with cross-border surveillance strategies in MENA.

## Conclusions

As asserted by the WHO Thirteenth General Programme of Work (2019–2023), SDG-3 is reliant on achieving UHC, addressing health emergencies, and promoting healthier populations [93]. Robust cross-border public health surveillance systems inclusive of mobile populations should be considered as fundamental to these three strategic priorities. The WHO HSS Building Block framework enabled a comprehensive approach to examine cross-border public health surveillance functionality in MENA and identify areas for improvement. Cross-border surveillance relies on effective leadership and governance for coordination between countries. Health facilities play a critical role in timely data collection and reporting. Sustainable financing supports infrastructure and training, while a skilled health workforce executes the functions of surveillance actions. Access to essential medicines and diagnostic tools ensures rapid diagnosis and response during outbreaks, and robust information systems facilitate real-time communication and data exchange inside and between countries [94]. The resulting synthesis provides several pathways to strengthen cross-border public health surveillance to address the causes and consequences of delayed or inadequate responses to disease outbreaks with potential spread across borders across data collection, analysis, and sharing.

Cross-border surveillance is an emerging field with unique challenges in MENA, though many other regions of the world provide lessons in collecting, analyzing, and sharing health data across borders with high mobility. For example, the US-Mexico border has a functional operational protocol for binational communication and coordination on disease notification and outbreaks in place. The shared border across these large nations witness significant and frequent movement of people across it, driven by tourism, border region residents and expatriates, temporary workers, and trade, as well as fleeing violence and economic instability [95]. In 2023 alone, there were over 187 million northbound border crossings at official land ports of entry [95]. Due to the constant travel between the border region and binational communities, the risk of spreading infectious diseases is increased, prompting the foundation of specific protocols. The Operational Protocol aligns with existing communication and outbreak procedures in local and state jurisdictions on both sides of the U.S.-Mexico border. It harmonizes with these procedures without substituting, duplicating or modifying any state or national policies, regulations, or standard operating procedures. It captures the consensus recommendations of the Binational Technical Working Group for effective binational collaboration, such as specifying the notifiable conditions, which align with IHR, the communication protocols, including focal points for each level of government, and the variables to be shared for binational case notification, which excludes any PHI of the individuals [96]. While the Gulf CDC’s improved communication and information sharing is one promising approach in MENA, the protocol from the US-Mexico Operational Protocol is another concrete example of how neighboring countries in MENA can consider operationalizing the IHR, while ensuring standardization and protection of PHI.

The WHO HSS framework can assist countries in responding to their obligations under the IHR, including for cross-border collaboration and communication. Inadequate cross-border surveillance systems lead to delayed or inadequate responses to disease outbreaks, resulting in excess morbidity and mortality. Thus, the WHO HSS Building Block framework application to IHR and cross-border surveillance has potential to expedite equitable progress towards SDG-3 in MENA. Mobile populations are diverse across countries and have varied reasons for mobility, among other characteristics. These differences currently account for many populations being excluded from systems of surveillance and health care, including informal traders, refugees, and internally displaced persons. As a result, these populations experience greater morbidity and mortality than formal, economically advantaged populations. Moreover, the disease dynamics of the mobile populations would directly or indirectly impact the receiving countries' populations, expanding their overall risks to diseases. Ensuring that all building blocks respond to all types of mobile populations will be critical, regardless of country income level or context. Consequently, inclusive, stronger surveillance and health systems will capture more complete information that will be vital to share with neighboring countries and international entities for strengthened cross-border surveillance and global healthy security.

In summary, applying the WHO HSS Building Blocks framework enabled a systematic method to translate our findings into recommendations on cross-border disease surveillance inclusive for all types of mobile populations in MENA. By evaluating the health system pillars, we identified strengths, challenges, and opportunities for improvement that could improve health security and resilience in MENA. This review highlights the importance of exploring and strengthening surveillance infrastructure and collaborative frameworks to safeguard health security and ensure equitable access to healthcare for all.

## Supplementary Information

Supplementary Material 1.

Supplementary Material 2.

## Data Availability

The qualitative data on which this article is based cannot be shared publicly due to confidentiality of the interviewees. The data analyzed for the manuscript are available from the corresponding author on reasonable request.

## Abbreviations

AI: Artificial Intelligence
CDC: Centers for Disease Control and Prevention
CDC STARS: Centers for Disease Control and Prevention Science, Technology, and Advanced Research Services
CEBS: Community Event-Based Surveillance
COVID-19: Coronavirus Disease of 2019
EMR: Electronic Medical Records
EMRO: Eastern Mediterranean Regional Office
EWARS: Early Warning and Response System
GCC: Gulf Cooperation Council
HIS: Health Information System
HSS: Health Systems Strengthening
IDIs: In-Depth Interviews
IDPs: Internally Displaced Persons
IHR: International Health Regulations
IOM: International Organization for Migration
IRB: Institutional Review Board
JEE: Joint External Evaluation
KSA: Kingdom of Saudi Arabia
MECIDS: Middle East Consortium for Infectious Disease Surveillance
MENA: Middle East and North Africa
MERS: Middle East Respiratory Syndrome
OECD: Organization for Economic Co-operation and Development
PHEIC: Public Health Emergency of International Concern
POE: Points of Entry
WHO: World Health Organization

## Footnotes

1 MENA country lists were defined by any country listed by WHO EMRO, World Bank MENA Region, or OECD MENA Region: Afghanistan, Algeria, Bahrain, Djibouti, Egypt, Iran, Iraq, Israel, Jordan, Kuwait, Lebanon, Libya, Morocco, Oman, Occupied Palestinian Territory, Palestinian Authority, Pakistan, Qatar, Saudi Arabia, Somalia, Sudan, Syria, Tunisia, UAE, West Bank & Gaza, Yemen. Malta & Mauritania were removed because of frequent inclusion in other regions.
