# Supplementary material for: Using the WHO building blocks to examine cross-border public health surveillance in MENA
Source: Int J Equity Health. 2025 Feb 6;24:38. doi: 10.1186/s12939-025-02393-7 (PMC11800598; doi:10.1186/s12939-025-02393-7)
Supplement: Supplementary file 1 — Supplementary Material 1. [file 12939_2025_2393_MOESM1_ESM.docx]

**Surveillance for Mobile Populations Landscape Analysis: Middle East North Africa (MENA) region**

**Background & Purpose:** University of California San Francisco (UCSF), in collaboration with US-CDC and WHO EMRO, are conducting a landscape analysis of public health/disease surveillance that includes mobile populations and the systems in place to support this surveillance and cross-border information sharing in the Middle East and North Africa (MENA). We wish to understand how these systems and structures can be improved following the COVID-19 pandemic and the current mpox outbreak, both of which spread outside of the countries in which they originated via travel. The purpose of this work is to understand the state of cross-border, regional, national, and point of entry public health/disease surveillance for these populations and the context around movement in the MENA region. The findings of this work will be used to inform countries’ allocation of existing resources and guide future engagement to strengthen cross-border surveillance, information sharing, and multi-sectoral collaboration in MENA aimed at improving global public health and pandemic preparedness.

**Methodology:** This landscape analysis includes two phases, a literature review and in-depth interviews

1. Literature Review: Literature review is being conducted of academic and grey literature to explore the state of existing cross-border, regional, national, and POE surveillance for mobile populations in MENA.
2. In-depth Interviews (IDI): We will conduct IDIs to explore perspectives of leaders and managers of key stakeholders, including international organizations and national Ministries of Health, Agriculture, and other departments responsible for border health and/or surveillance, including International Health Regulations national focal points. We will first conduct the interviews at the regional/international level followed by country level for 2 to 5 countries. The team will purposively select the countries based on findings from the literature review, regional interviews, and comments from the collaborators.

**In-depth Interview Procedures:** Participation is voluntary and will involve one in-depth interview that will last roughly one hour, either virtually on zoom or in person. Interviews will be conducted in English, Arabic, or French. The interviews will be conducted in a private location if in person or in a password protected virtual meeting. Privacy will also be protected in several ways: 1) no participant will be identified in any report or publication; 2) all project materials and data collection forms will be identified by type of organization and role of the participant only; 3) notes from interviews will be kept in locked offices and/or in a locked file cabinet or password protected computer; 4) data will be analyzed collectively and individual participant data will remain anonymous. 5) password protection will be assured. Data will be stored securely on password-protected computers and networks encrypted and maintained by the team. Electronic data will be protected with a password.

**Dissemination:** Information will be summarized from the literature review and from the interviews to summarize overarching themes surrounding cross-border disease surveillance and provide recommendations intended to improve outcomes and create an enabling environment for cross-border surveillance and border health. A policy brief/summary report will be produced and results may be published in international peer- reviewed journals. Results may be presented at national and international policy meetings and/or conferences, as well as a dissemination workshop for key stakeholders in MENA.

**Questions about the project:** If you have questions, the program team is immediately available. If you have any questions after you have completed your interview you can reach the program coordinator:

*MENA Program Manager:* Farah Massoud

*Email:* [*Farah.massoud@ucsf.edu*](mailto:Farah.massoud@ucsf.edu)

**تحليل وضع الترصد للسكان المتنقلين في منطقة الشرق الأوسط وشمال إفريقيا (MENA)**

**الخلفية والغرض:** تجري جامعة كاليفورنيا سان فرانسيسكو (UCSF) بالتعاون مع US-CDC ومنظمة الصحة العالمية – المكتب الإقليمي لشرق المتوسط تحليل وضع ترصد الصحة العمومية والأمراض التي تشمل السكان المتنقلين والأنظمة الموجودة لدعم الترصد وتبادل المعلومات عبر الحدود في الشرق الأوسط وشمال إفريقيا (MENA) . تحليل الوضع سيوضح كيفية تحسين هذه الأنظمة والهياكل بعد جائحة كورونا وفاشية جدري القردة الحالية حيث أن كلاهما انتشر خارج البلدان التي نشآ فيها عن طريق السفر. الغاية من العمل هو فهم حالة الترصد الوطنية والإقليمية للصحة العمومية والأمراض العابرة للحدود ونقاط الدخول للسكان المتنقلين والسياق حول التنقل في منطقة الشرق الأوسط وشمال إفريقيا. سيتم استخدام نتائج هذا التحليل لإعلام الدول بتخصيص الموارد الحالية وتوجيه العمل المستقبلي لتعزيز الترصد عبر الحدود وتبادل المعلومات والتنسيق المتعدد القطاعات في منطقة الشرق الأوسط وشمال إفريقيا بهدف تحسين الصحة العامة على الصعيد العالمي والتأهب للأوبئة.

**المنهجية:** يتضمن هذا التحليل مرحلتين: مراجعة الدراسات السابقة والمقابلات الشخصية المطولة

1. **مراجعة الدراسات السابقة:** يتم إجراء مراجعة الدراسات الأكاديمية والمنشورات الغير رسمية لاستكشاف حالة الترصد الوطنية والإقليمية والترصد عبر الحدود ونقاط الدخول للسكان المتنقلين في منطقة الشرق الأوسط وشمال إفريقيا.
2. **المقابلات الشخصية المطولة**: سيتم اجراء المقابلات الشخصية المطولة لاستكشاف وجهات نظر أصحاب المصلحة الرئيسيين بما في ذلك المنظمات الدولية والوزارات الوطنية مثل وزارة الصحة والزراعة واي قطاعات أخرى مسؤولة عن الصحة عبر الحدود، بما في ذلك ضباط الاتصال الوطنيون المعنيون باللوائح الصحية الدولية. أولا ستجرى المقابلات على المستوى الإقليمي والدولي يليها المستوى الوطني في 2 إلى 5 دول. سيختار الفريق البلدان على أساس النتائج من مراجعة الدراسات السابقة والمقابلات الإقليمية والملاحظات من جميع الشركاء.

**إجراءات المقابلات الشخصية المطولة:** المشاركة طوعية وستتضمن مقابلة واحدة متعمقة تستمر لمدة ساعة تقريبًا، إما عبر الإنترنت عن طريق تطبيق Zoom أو بشكل شخصي. سيتم إجراء المقابلات باللغة الإنجليزية أو العربية أو الفرنسية. سيتم إجراء المقابلات في مكان خاص إذا كان ذلك شخصيًا أو في اجتماع عبر الإنترنت عن طريق تطبيق Zoom محمي بكلمة مرور.

ستتم حماية الخصوصية بعدة طرق: 1) لن يتم تحديد هوية أي مشارك في أي تقرير أو منشور 2) سيتم تحديد جميع مواد المشروع واستمارات جمع البيانات حسب نوع المنظمة ودور المشارك فقط 3) سيتم الاحتفاظ بالملاحظات من المقابلات في مكاتب مقفلة و / أو في خزانة ملفات مقفلة أو كمبيوتر محمي بكلمة مرور 4) سيتم تحليل البيانات بشكل جماعي وستظل بيانات المشاركين الفردية مجهولة 5) سيتم ضمان حماية كلمة المرور. البيانات ستخزن بشكل آمن على أجهزة الكمبيوتر والشبكات المحمية بكلمة مرور والتي يتم تشفيرها وصيانتها بواسطة الفريق. ستتم حماية البيانات الإلكترونية بكلمة مرور.

**نشر نتائج البحث:** سيتم تلخيص المعلومات المتاحة من مراجعة الدراسات السابقة ومن المقابلات لتلخيص الموضوعات الشاملة المحيطة بترصد الأمراض عبر الحدود وتقديم توصيات تهدف إلى تحسين النتائج وخلق بيئة مواتية للترصد عبر الحدود. سيتم إعداد تقرير موجز وقد يتم نشر النتائج في المجلات الدولية الأكاديمية/العلمية. يمكن تقديم النتائج في اجتماعات السياسات الوطنية والدولية و / أو المؤتمرات بالإضافة إلى ورشة عمل لنشر النتائج لأصحاب المصلحة الرئيسيين في منطقة الشرق الأوسط وشمال إفريقيا.

**أسئلة حول المشروع:** سيكون فريق البرنامج متاحًا على الفور في حال وجود أي أسئلة. إذا كان لديك أي أسئلة بعد الانتهاء من المقابلة، يمكنك التواصل مع مديرة البرامج: فرح مسعود

الايميل: [farah.massoud@ucsf.edu](mailto:farah.massoud@ucsf.edu)
