# Supplementary material for: Using the WHO building blocks to examine cross-border public health surveillance in MENA
Source: Int J Equity Health. 2025 Feb 6;24:38. doi: 10.1186/s12939-025-02393-7 (PMC11800598; doi:10.1186/s12939-025-02393-7)
Supplement: Supplementary file 2 — Supplementary Material 2. [file 12939_2025_2393_MOESM2_ESM.docx]

**Supplement 1: In-Depth Interview Guides**

| **Regional Level In-Depth Interview Guide** | |
| --- | --- |
| **Estimated Time: one hour** | |
| **Country Code:** | **Informant Code:** |
| **Interviewer Name:** | **Notetaker Name:** |
| **Project Background/Objectives and Verbal Consent**  *Introduction of Interviewer/team:* names, positions, agencies.  We, on behalf of the University of California San Francisco and US-CDC, are conducting a project on cross-border public health communicable disease surveillance. This specifically includes mobile populations and the systems in place to support this surveillance and cross-border information sharing in the Middle East and North Africa region, or MENA. This project is intended to benefit public health systems because it will help us understand gaps and identify specific challenges. We also hope to identify best practices that currently exist within countries across the MENA region. We intend that these findings will be shared to further enhance cross-border surveillance functionality for future implementation. We are grateful for your time today.  I am interested in your experiences as *a ______ (regional/country level stakeholder/Ministry of Health/other official).*  We want to remind you that participating in this project is voluntary. You can choose not to participate, refuse to answer any questions, and stop at any time. Your privacy will be protected. Your identity or personal information will not be disclosed in any report, presentation, or publication that may result from the project. Notes that are taken during the interview will be stored in a secure location. With your permission, we will record the interview; we will delete the recording after we have made a transcript of what was said. This interview should not take more than one hour.  Agrees to be interviewed? Yes____ No____  Agrees to be recorded: Yes____ No ____  Do you have any questions before we begin? | |
| **Section A. Mobile Populations: situation analysis** | |
| A.1. How does the Eastern Mediterranean/MENA region define and characterize “mobile populations”?   - *Prompt if necessary: For example, travelers, voluntary immigrants, refugees, etc.* - Is there more than one definition used? - In your opinion, are there any challenges related to the definition (s) of mobile populations? *If yes, prompt to describe the challenges, especially related to varying/inconsistent definitions* - *Probe: Are there any efforts to harmonize definitions?* | |
| A.2. Tell us about data on mobile populations and mobility in the Eastern Mediterranean/MENA region?   - What are the main data sources? - What are the primary indicators? How are indicators defined? Are there regional definitions? - Who is responsible for collecting/analyzing/reporting this data?   - Probe: roles of different partners?   - Probe: data integrated into national systems? - What are the gaps in quality/availability of data? (*Prompt: Explore if gaps differ by mobile population type)* - Have you ever used or considered using data from social media platforms, wearable devices, or mobile phones? Why or why not? - What data is collected at different points of entry? | |
| A.3. Mobile populations in MENA are very diverse, including linguistically and culturally. How do the systems in MENA navigate the cultural sensitivities/linguistic diversity/vulnerabilities of collecting public health data from mobile populations? | |
| A.4 What data would you like to see collected at the national or regional level, but may not have the infrastructure to do so yet? | |
| **Section B. Cross-border surveillance systems & policies** | |
| B.1. What are the different approaches in the Eastern Mediterranean/MENA region to public health/disease surveillance that include mobile populations and how are they functioning?   - Which approaches are effective? Why? - Which approaches are less effective? Why? - Are different approaches used in emergency or routine settings? - In your opinion, do current surveillance modalities serve some mobile populations or points of entry better than others; if so, why?   - Does surveillance at some points of entry’s function better than others? If so, why?   - Does surveillance for certain diseases function better than others? If so, why? - *Probe: is data on mobile populations part of national HIS/surveillance systems or separate?* | |
| B.2. What are the existing policies and legal frameworks at the **regional/international level** related cross-border surveillance and mobile population health, including cross-border information sharing and regional coordination?   - In your opinion, how do these policies impact the effectiveness of cross-border surveillance? - In your opinion, do you suggest any new policies or adaptations to existing policies to better address cross-border public health surveillance needs? - *Probes: IHR/IHR Focal Points functionality* | |
| B.3. Tell us about the experiences in the Eastern Mediterranean/MENA region with neighboring countries in terms of coordination and information sharing for cross-border surveillance:   - Are there different approaches or challenges with various neighboring countries? - Are there any examples of strong coordination or information sharing, i.e. in certain outbreaks (COVID/MPOX) amongst certain countries? - *Probe: Are Data sharing agreements in place/useful? Are case definitions shared or harmonized?* | |
| B.4. What are the existing policies and legal frameworks at the **national level** related to cross-border surveillance and mobile population health?   - In your opinion, how do these policies impact *(prompt: facilitate or impede)* the effectiveness of cross-border surveillance? - In your opinion, do you suggest any new policies or adaptations to existing policies to better address cross-border public health surveillance needs? - Probes*: IHR/IHR Focal Points functionality* | |
| B.5. **International Health Regulations: Additional probes if not covered**  Please describe how the IHR recommendations are currently put into place in Eastern Mediterranean/MENA countries:   - What technical areas of the IHR are implemented most thoroughly in MENA? - How does your organization support countries to implement IHR? - What countries are strongest implementing IHR? - What challenges/gaps exist to implementing the IHR in MENA? - How do recommend addressing these challenges/gaps?   - *Probe: Revised guidelines? Human resources? Technical Support?* | |
| **Section C. Multi-sectoral/multi-stakeholder collaborations & One Health** | |
| C.1. Tell us about the multi-sectoral collaboration around mobile populations and cross-border surveillance in MENA/EM at the regional/international level?   - What sectors are involved in cross-border surveillance? - What characteristics make multisectoral collaborations work effectively towards achieving the identified goals? - What challenges exist to sustaining multisectoral collaborations? - Do you have any examples of “successful” multisectoral collaboration that strengthened border health systems or disease surveillance? *(probe, perhaps specific to a certain disease/outbreak or certain country)* | |
| C.2. In addition to various sectors, tell us about multi-stakeholder collaboration/Task Forces in within the region:   - What characteristics make multi-stakeholder collaborations/Task Forces work effectively towards achieving the identified goals? - What challenges exist to sustaining multi-stakeholder collaborations/Task Forces collaborations? - Do you have any examples of “successful” multi-stakeholder/Task Force collaboration that strengthened border health systems or disease surveillance? *(probe, perhaps specific to a certain disease/outbreak)*   - *Probe: Regional Taskforce on COVID-19 and Mobility/Migration* | |
| C.3. Tell us about your approach to One Health in MENA/EM as it relates to cross-border surveillance and mobile populations:   - What is needed to integrate this approach? - What are the biggest challenges to your adopting the One Health approach? - Are there any countries with formal connections between public health administration and animal/veterinary health sectors working well? | |
| C.4 Tell us about resource allocation within multi-sectoral approaches and cross-border surveillance at the regional level in MENA:   - What challenges exist? - Are there significant differences in resources allocated to different sectors that impact cross-border surveillance? | |
| **Section D. Outbreaks/Case Studies** | |
| D.1. Reflecting on recent outbreaks, such as COVID-19, MERS-CoV, hMPXV, tell us about how cross-border public health information exchange functioned in regional response?   - What worked well? Why? - What did not work well? Why? - What lessons were learned with respect to risks posed by population mobility? Is anything being done to address this? | |
| D.2. Case Studies: What countries have implemented effective cross-border surveillance approaches in recent outbreaks/pandemics that you would recommend highlighting?  *Probe on existing possibilities:*   - Egypt/Sudan/Libya - Oman/Yemen/Saudi Arabia - Jordan/Lebanon/Syria | |
| **Closing:**  Do you have anything else to add?  Do you have any questions for us?  Is there anyone else you recommend we interview?  Thank you for your time, we will be sure to share findings and consult with you for any additional questions. | |

| **National Level Stakeholder In-Depth Interview Guide** | |
| --- | --- |
| **Estimated Time: one hour** | |
| **Country Code:** | **Informant Code:** |
| **Interviewer Name:** | **Notetaker Name:** |
| **Landscape Analysis Background/Objectives and Verbal Consent**  *Introduction of Interviewer/team:* names, positions, agencies.  We, on behalf of the University of California San Francisco and US-CDC, are conducting a landscape analysis on cross-border public health communicable disease surveillance. This specifically includes mobile populations and the systems in place to support this surveillance and cross-border information sharing in the Middle East and North Africa region, or MENA. This analysis is intended to benefit public health systems because it will help us understand gaps and identify specific challenges. We also hope to identify best practices that currently exist within countries across the MENA region. We intend that these findings will be shared to further enhance cross-border surveillance functionality for future implementation. We are grateful for your time today.  I am interested in your experiences as *a ______ (regional/country level stakeholder/Ministry of Health/other official).*  We want to remind you that participating in this project is voluntary. You can choose not to participate, refuse to answer any questions, and stop at any time. Your privacy will be protected. Your identity or personal information will not be disclosed in any report, presentation, or publication that may result from the project. Notes that are taken during the interview will be stored in a secure location. With your permission, we will record the interview; we will delete the recording after we have made a transcript of what was said. This interview should not take more than one hour.  Agrees to be interviewed? Yes____ No____  Agrees to be recorded: Yes____ No ____  Do you have any questions before we begin? | |
| **Section A. Mobile Populations: Situation Analysis** | |
| A.1. How does your country define and characterize “mobile populations” ?   - *Prompt if necessary: For example, travelers, voluntary immigrants, refugees, etc.* - Is there more than one definition used? - In your opinion, are there any challenges related to the definition (s)?   - *If yes, prompt to describe the challenges. Prompt any challenges about multiple or inconsistent definitions* | |
| A.2. Tell us about your data on mobile populations and mobility?   - What are your main data sources? - What are the primary indicators? How are indicators defined? - Who is responsible for collecting/analyzing/reporting this data?   - Probe: roles of different partners?   - Probe: data integrated into national systems? - What are the gaps in quality/availability of data? (*Prompt: Explore if gaps differ by mobile population type)* - Have you ever used or considered using data from social media platforms, wearable devices, or mobile phones? Why or why not? - What data is collected at different points of entry? | |
| A.3. Mobile populations in MENA are very diverse, including linguistically and culturally. How does your system navigate the cultural sensitivities/linguistic diversity/vulnerabilities of collecting public health data from mobile populations? | |
| A.4 What data would you like to collect, but may not have the authority or infrastructure to do so yet? | |
| **Section B. Cross-border surveillance systems & policies** | |
| B.1. What are the different approaches in your country to public health/disease surveillance that include mobile populations and how are they functioning?   - Which approaches are effective? Why? - Which approaches are less effective? Why? - Are different approaches used in emergency or routine settings? - In your opinion, do current surveillance modalities serve some mobile populations or points of entry better than others; if so, why?   - Does surveillance at some points of entry’s function better than others? If so, why?   - Does surveillance for certain diseases function better than others? If so, why? - *Probe: is data on mobile populations part of national HIS/surveillance systems or separate?* | |
| B.2. What are the existing policies and legal frameworks at the **national level** related to cross-border surveillance and mobile population health?   - In your opinion, how do these policies impact *(prompt: facilitate or impede)* the effectiveness of cross-border surveillance? - In your opinion, do you suggest any new policies or adaptations to existing policies to better address cross-border public health surveillance needs? - Probes: IHR/IHR Focal Points functionality | |
| B.3. Tell us about your experiences with neighboring countries in terms of coordination and information sharing for cross-border surveillance:   - Are there different approaches or challenges with various neighboring countries? - Are there any examples of strong coordination or information sharing, i.e. in certain outbreaks (COVID/MPOX) - *Probe: Are Data sharing agreements in place/useful? Are case definitions shared or harmonized?* | |
| B.4. What are the existing policies and legal frameworks at the **regional/international level** related cross-border surveillance and mobile population health, including cross-border information sharing and regional coordination?   - In your opinion, how do these policies impact the effectiveness of cross-border surveillance? - In your opinion, do you suggest any new policies or adaptations to existing policies to better address cross-border public health surveillance needs? - Probes: *IHR/IHR Focal Points functionality* | |
| B.5. **International Health Regulations** (optional, depending on respondent)  Please describe how the IHR recommendations are currently put into place in your country:   - What technical areas of the IHR is your country strongest in? - What challenges/gaps exist to implementing the IHR? - Is there any collaboration with other sectors to implement the IHR? *If yes,* please explain. - Are any ground crossings designated for developing IHR capacities? *If yes, probe:*   - What are the surveillance systems in place for these borders?   - At these borders, has your country entered into any bilateral/multilateral agreements with neighboring countries? | |
| **Section C. Multi-sectoral/multi-stakeholder collaborations & One Health** | |
| C.1. Tell us about the multi-sectoral collaboration around mobile populations and cross-border surveillance in your country?   - What sectors are involved in cross-border surveillance? - Are collaborations within your country or international? - What characteristics make multisectoral collaborations work effectively towards achieving the identified goals? - What challenges exist to sustaining multisectoral collaborations? - Do you have any examples of “successful” multisectoral collaboration that strengthened border health systems or disease surveillance? *(probe, perhaps specific to a certain disease/outbreak)* | |
| C.2. In addition to various sectors, are there any other key stakeholders that impact cross-border surveillance? Tell us about multi-stakeholder collaboration/Task Forces in your country or internationally within the region:   - *Probe: Collaboration with WHO EMRO, IOM, the United Nations Economic and Social Commission for Western Asia, and the International Labour Organization under the United Nations Inter-Agency Issue-based Coalition/ Regional Taskforce on COVID-19 and Mobility/Migration* - What characteristics make multi-stakeholder collaborations/Task Forces work effectively towards achieving the identified goals? - What challenges exist to sustaining multi-stakeholder collaborations/Task Forces collaborations? - Do you have any examples of “successful” multi-stakeholder/Task Force collaboration that strengthened border health systems or disease surveillance? *(probe, perhaps specific to a certain disease/outbreak)* | |
| C.3. Tell us about your approach to One Health in your country as it relates to cross-border surveillance and mobile populations:   - Are there any formal connections between public health administration and animal/veterinary health in your country? - What is needed to integrate this approach? - What are the biggest challenges to your country adopting the One Health approach? | |
| C.4 Tell us about resource allocation within multi-sectoral approaches and cross-border surveillance:   - What challenges exist? - Are there significant differences in resources allocated to different sectors that impact cross-border surveillance?   - Probe: National vs. regional challenges/approaches | |
| **Section D. Outbreaks/Case Studies** | |
| D.1. Reflecting on recent outbreaks, such as COVID-19, MERS-CoV, hMPXV, tell us about how cross-border public health information exchange functioned in your national/regional response?   - What worked well? Why? - What did not work well? Why? - What lessons were learned with respect to risks posed by population mobility? Is anything being done to address this? | |
| **Closing:**  Do you have anything else to add?  Do you have any questions for us?  Is there anyone else you recommend we interview?  Thank you for your time, we will be sure to share findings and consult with you for any additional questions. | |
